# The role of Glut-1 and H^+^/K^+^-ATPase expression in hyperplasia of mice laryngeal epithelium induced by pepsin

**DOI:** 10.1007/s00405-021-07221-6

**Published:** 2022-01-27

**Authors:** Yin-Jie Ao, Ting-Ting Wu, Zai-Zai Cao, Shui-Hong Zhou, Yang-Yang Bao, Li-Fang Shen

**Affiliations:** Department of Otolaryngology, The First Affiliated Hospital, College of Medicine, Zhejiang University, 79 Qingchun Road, Hangzhou, 310003 People’s Republic of China

**Keywords:** Laryngopharyngeal reflux, Experimental animal models, Pepsin, Laryngeal mucosa, Hyperplasia

## Abstract

**Purpose:**

To explore the role played by Glut-1 and H^+^/K^+^-ATPase in pepsin-induced, mouse laryngeal epithelial proliferation, growth, and development.

**Methods:**

We established a mouse model of laryngopharyngeal reflux and measured Glut-1 and H^+^/K^+^-ATPase expression levels in mouse laryngeal epithelium treated with artificial gastric juice containing pepsin.

**Results:**

Artificial pepsin-containing gastric juice induced significant hyperplastic changes in mouse laryngeal epithelium compared to control mice at 15, 30, and 45 days. Inhibition of Glut-1 expression by 2-DG significantly suppressed such hyperplasia compared to mice exposed to artificial gastric juice containing pepsin at 15, 30, and 45 days. After treatment with pepsin-containing artificial gastric juice, RT-PCR and Western blotting showed that the levels of Glut-1 and H^+^/K^+^-ATPase α, β increased significantly.

**Conclusions:**

Pepsin-containing artificial gastric juice promoted mouse laryngeal epithelial hyperplasia associated with abnormal expression of Glut-1 and H^+^/K^+^-ATPase α, β.

## Introduction

Laryngopharyngeal reflux (LPR) refers to the reflux of gastric contents above the upper esophageal sphincter, causing various symptoms and signs [1]. The gastric contents include gastric acid and non-acids such as pepsin, cholate, bacteria, and trypsin [2, 3]. Tissue damage caused by non-acidic components cannot be alleviated by proton pump inhibitors (PPIs) alone [3]. When acidic material briefly flows back to the laryngopharynx, the tissue damage caused by gastric acid is diminished by dilution in saliva and by the higher laryngopharyngeal pH4. Tissue damage caused by pepsin is an important pathogenetic factor [4]. Esophageal mucosal epithelial injury is caused principally by gastric acid secreted by the proton pump H + /K + -ATPase [3]; pepsin damages the laryngopharyngeal epithelium [4, 5]. Pepsin is abnormally expressed in patients with voice disorders, vocal cord polyps, laryngeal stenosis, vocal cord leukoplakia, and malignant lesions [5, 6]. We earlier reported high pepsin levels in vocal cord polyps and patients with vocal cord leukoplakia [5]; the latter expression level increased significantly as the dysplasia grade rose [5]. We also found that high Glut-1 expression may improve the development of vocal cord leukoplakia by upregulating laryngeal H^+^/K^+^-ATPase expression to reactivate absorbed pepsin resulting in laryngeal mucosa injury in vitro [6]*.* There is the same mechanism of pepsin-mediated tissue damage in vivo is unclear. Pepsin activation is closely related to the microenvironmental pH and is highest at pH 1.5–3.0. As the pH increases, the activity gradually falls. Thus, at the pH of gastric acid, pepsin is highly active.

The principal function of the proton pump H + /K + -ATPase is gastric acid secretion. H + /K + -ATPases are present in other organs, including the larynx [4, 7–9], where they also mediate acid secretion [7, 10]. The resulting pH decrease re-activates absorbed refluxed pepsin, damaging the mucosa and triggering laryngeal inflammatory or carcinogenic changes [4, 9, 11, 12]. We earlier detected H + /K + -ATPase in normal laryngeal tissues and found that the expression thereof was higher in laryngeal carcinomas [8]. Therefore, we hypothesized that acid secreted via the action of the laryngeal mucosal H + /K + -ATPase altered the laryngeal microenvironment, re-activating pepsin and changing the laryngeal epithelium.

Traditionally, gastroesophageal reflux diseases are characterized by caustic chemical damage attributable to refluxed acid [8, 9]. However, some studies have found that reflux esophagitis reflects cytokine-mediated inflammation associated with hypoxia [13]. Hypoxia-inducible factor (HIF)-2α plays a major role in this process [14]. Hypoxia-induced transcription factors such as HIF-2α upregulate hypoxia-response genes in Barrett’s esophagus (BE) tissue, including that encoding the glucose transporter Glut-1 [15]. High-level expression of Glut-1 may increase glycolysis. H + /K + -ATPase may be associated with aerobic glycolysis (the Warburg effect) during intestinal metaplasia of the gastric corpus mucosa, as PI3K/AKT/mTOR signaling is activated [16] and then AKT directly increases the surface translocation of glucose transporters (including Glut-1), enhancing aerobic glycolysis [15]. In patients with LPRD, large amounts of energy are required during H + secretion by H + /K + ATPase. We speculate that both glycolysis and oxidative phosphorylation play roles in this process. On this basis, we hypothesized that Glut-1 played an important role in pepsin-induced laryngeal mucosal damage.

To test these hypotheses, we established a mouse model of reflux and investigated the association thereof with the proliferation, growth, and development of mouse laryngeal epithelium. We also explored the roles played by Glut-1 and H + /K + -ATPase in these processes.

## Materials and methods

This study was approved by the experimental animal ethics committee of the First Affiliated Hospital, College of Medicine, Zhejiang University, China (approval no. 202103). All authors had access to all study data and reviewed and approved the final manuscript. The study conformed to the Declaration of Helsinki.

### Mouse model of LPR

This study was conducted in accordance with the ethical standards of the National Research Council Guide for the Care and Use of Laboratory Animals. Healthy male C57BL/6b mice (4 weeks of age, 18–20 g) were purchased from Shanghai Slake Laboratory Animal Co., Ltd. (license no. SCXK [Shanghai] 2017-0005). We modified an established mouse model of LPR [16]; the larynx and hypopharynx were exposed once daily to 200-μL amounts of the solutions indicated below using a syringe fitted with an irrigating needle. The mice were divided into six experimental and three control groups (10 mice/group). Control mice were exposed to saline at pH 7 for 15, 30, and 45 days. Experimental mice were exposed to artificial gastric juice containing pepsin at pH 2 for 15, 30, and 45 days; or to artificial gastric juice containing pepsin with 2-deoxyglucose (2-DG; [5 mg/500 mL] to inhibit Glut-1 expression) at pH 2 for 15, 30, and 45 days. The mice were euthanized and the larynges removed under a microscope. The dissected larynges of five mice/group were immediately transferred to 10% (v/v) neutral buffered formalin for embedding in paraffin blocks. The larynges of the remaining five mice per group were immediately placed at − 80 °C prior to reverse transcriptase–polymerase chain reaction (RT-PCR) and Western blotting.

### Histological evaluation

Approximately 8-μm-thick sections of formalin-fixed paraffin-embedded laryngeal tissues were stained with hematoxylin-and-eosin and visualized under a light microscope. We sought hemorrhagic or ulcerative lesions that could indicate local toxicity attributable to the artificial gastric juice.

### Quantitative real-time RT-PCR

Total RNA was isolated using EZB-RT2GQ SYBR ON according to the manufacturer’sinstructions. Briefly, 1 µg amounts of RNA were reverse-transcribed using a First-Strand cDNA Kit and subjected to PCR using a SYBR Green qPCR Kit, incubated at 42 ℃ for 15 min, and stored at − 20 ℃. RNA primers were designed and synthesized by Sangon. The primers for Glut-1 (Abcam, Cambridge, UK) were forward 5′-GGTCATGAGTATGGCACAACC-3′ and reverse 5′-GTCAACACGGCCTTCAC-3′. The primers for H^+^/K^+^-ATPase α (Abcam) were forward 5′-CATCATCGCCAGCTTTAAGAAC-3′ and reverse 5′-CAGCGTTGATCTGGAATTTGTC-3′. The primers for H^+^/K^+^-ATPase β (Abcam,) were forward 5′-CAGTCTGCACTACTTCCCTTAT-3′ and reverse 5′-CACTTTCCCTTCATACGGGTC-3′. The primers for anti-glyceraldehyde 3-phosphate dehydrogenase (GAPDH) (CST, Boston, MA, USA) were forward 5′-GAAGGTGAAGGTCGGAGTC-3′ and reverse 5′-GAAGATGGTGATGGGATTTC-3′. The PCR products were 111 bp (Glut-1), 80 bp (α-subunit), 175 bp (β-subunit), and 172 bp (GAPDH) in length. The 2^ΔΔCt^ method was used to calculate relative gene expression levels. All experiments were performed in triplicate.

### Western blotting

Western blotting was performed in accordance with the manufacturer’s instructions (Abcam). Rabbit monoclonal anti-Glut-1 (1:1,000), -H^+^/K^+^-ATPase α (1:2000), and -H^+^/K^+^-ATPase β (1:1000) were purchased from Abcam; and -GAPDH (1:1000) from CST. The secondary antibodies were goat anti-rabbit antibodies (1:1000) conjugated with horseradish peroxidase (Abcam). Signals were visualized using ImageJ software (National Institutes of Health, Bethesda, MD, USA). Protein levels were quantified by scanning densitometry (in triplicate).

### Immunohistochemistry

Paraffin sections were cut at a thickness of 5 µm. After deparaffinization and hydration, the sections underwent antigen retrieval using the microwave oven method. Endogenous peroxidase was blocked in 3% (v/v) H_2_O_2_ for 25 min at room temperature. Next, the slides were incubated with primary antibodies against Glut-1 (1:200), H^+^/K^+^-ATPase α (1:200), and H^+^/K^+^-ATPase β (1:200) overnight. The next day, the sections were incubated with the corresponding secondary antibodies (1:200) at room temperature for 50 min, stained using a DAB Staining Kit, and subjected to hematoxylin-and-eosin staining. The sections were photographed under a microscope; cells that contained brownish-yellow granules were considered positive. Five high-magnification fields (× 400) were randomly selected, in each of which 100 cells were counted; scoring was as follows: 0; 1, < 25%; 2, 26–50%; and 3, ≥ 50% positive cells. Dye depth was scored as follows: 0, no staining; 1, light yellow; 2, brownish-yellow; and 3, deep brownish-yellow. The immunohistochemical score was the positive-cell score + the dye-depth score. Total scores of 0–1, 2, 3–4, and 5–6 were considered negative (–), weakly positive ( +), positive (+ +), and strongly positive (+ + +), respectively. The investigator was blinded to group allocation.

### Statistical analysis

The associations of Glut-1 and H^+^/K^+^-ATPase expression with laryngeal epithelial damage were assessed using the chi-squared test or Fisher exact test. Continuous data are expressed as means ± standard deviations and were compared employing the dependent *t* test when exploring within-subject differences. We performed Pearson correlation analysis. *P* values < 0.05 were indicative of statistical significance. GraphPad Prism 7 software was used to draw graphs and statistical analysis was performed with the aid of SPSS Statistics for Windows (ver. 19.0.; IBM Corp., Armonk, NY, USA).

## Results

### Pepsin induces laryngeal epithelial hyperplasia

**Fig. 1:**
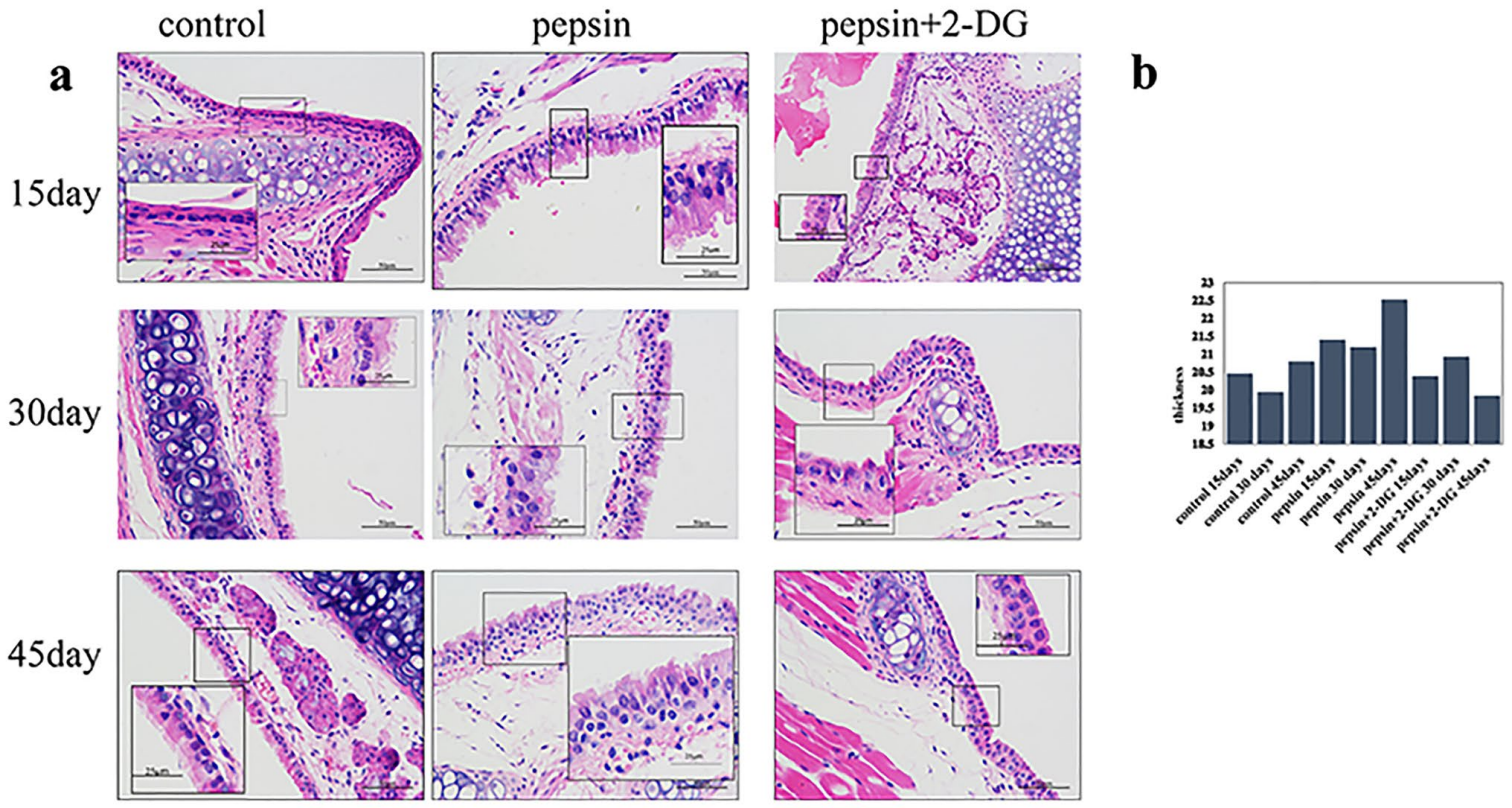
Changes in mouse laryngeal epithelium after exposure to pepsin-containing artificial gastric juice (**a**) and inhibition of Glut-1 expression by 2-DG (**b**)

Artificial gastric juice containing pepsin induced significant hyperplastic alterations in mouse laryngeal epithelium compared to control mice at 15, 30, and 45 days (*p* < 0.05; Fig. 1); however, the effect did not increase over time. Inhibition of Glut-1 expression by 2-DG significantly suppressed such hyperplasia compared to mice exposed to artificial gastric juice containing pepsin for 15, 30, and 45 days (*p* < 0.05; Fig. 1).

### Pepsin increases Glut-1 and H^+^/K^+^-ATPase expression in mouse hyperplastic laryngeal epithelium

**Fig. 2:**
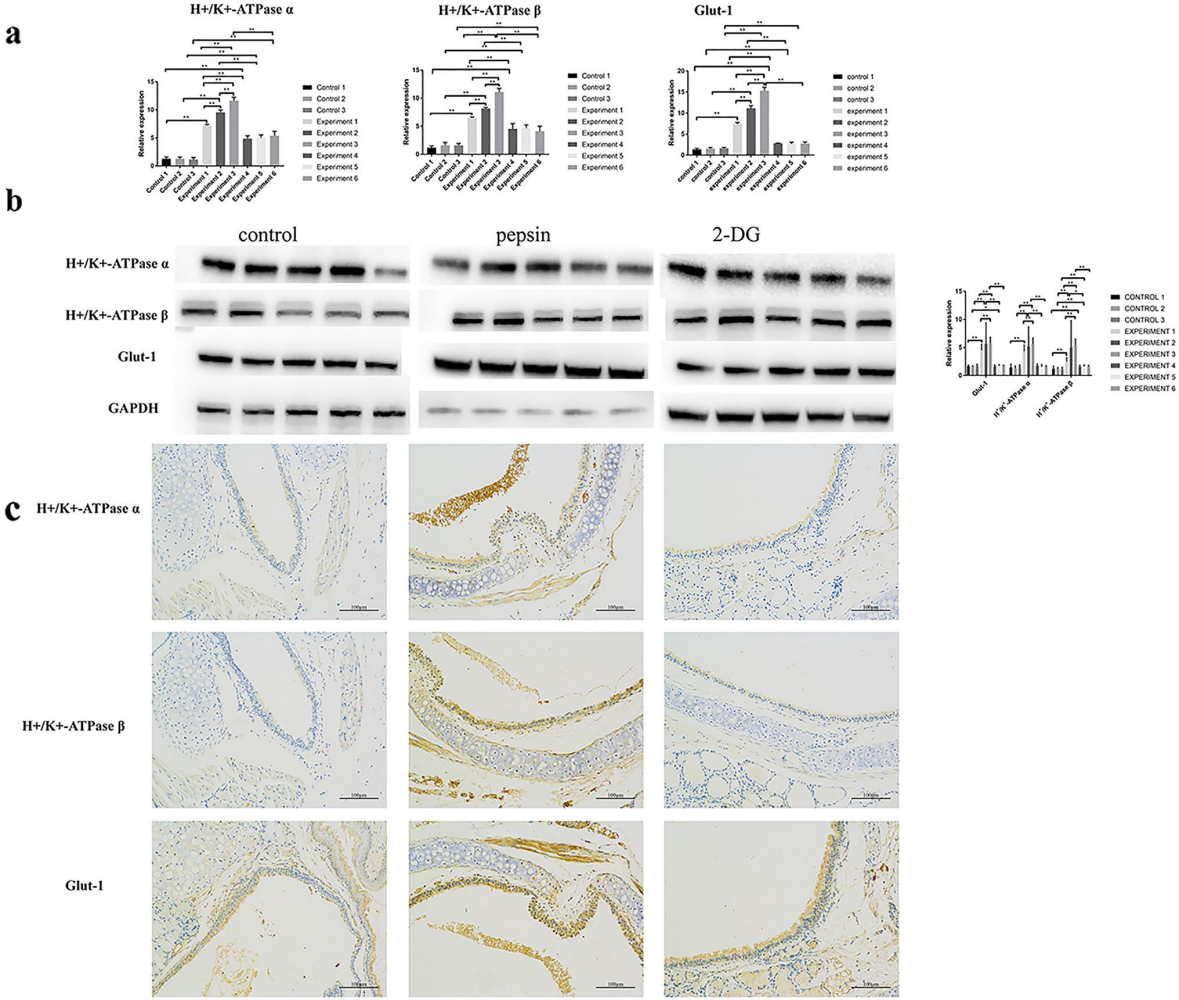
Glut-1 and H^+^/K^+^-ATPase α, β expression levels in mouse laryngeal epithelium exposed to pepsin-containing artificial gastric juice and 2-DG as revealed by **a** RT-PCR and **b** Western blotting

Artificial gastric juice containing pepsin significantly increased the Glut-1 and H^+^/K^+^-ATPase α, β mRNA and protein levels in mouse hyperplastic laryngeal epithelium, in a time-dependent manner (both *p* < 0.05, Fig. 2). In contrast, pepsin-plus-2-DG significantly decreased the Glut-1 and H^+^/K^+^-ATPase α, β mRNA and protein levels in a time-dependent manner (both *p* < 0.05, Fig. 2). Immunohistochemistry showed that 2-DG significantly inhibited H^+^/K^+^-ATPase α, β, but not Glut-1, expression (*p* < 0.05, Fig. 2). A positive correlation was evident between Glut-1 and H^+^/K^+^-ATPase α, β expression.

## Discussion

LPR disease (LPRD) is considered to be the extraesophageal manifestation of gastroesophageal reflux disease (GERD) [17]. As it is difficult to study the pathophysiology of LPR in humans, LPR animal models are very useful [18]. Several GERD models are available, but few LPRD models. Hu et al. established a rabbit reflux model employing total cardiomyectomy [19]. The limitations include a difficult operation, the need for postoperative care, and animal morbidity [19, 20]. The mucosal epithelium of the human larynx is principally nonkeratinized, stratified squamous epithelium [19, 20]. The laryngeal mucosal epithelium of pigs and dogs is very similar, but these animals are expensive, their nutrition is complex, and it difficult to perform large-scale experiments [20]. Lou et al. used nasogastric intubation when establishing a rabbit model of LPR; however; this was associated with deaths from aspiration pneumonia [21]. Sasaki et al. established a mouse model of LPR using a plastic feeding tube to administer bile or control fluids to the larynx [21]. This simulated reflux in the upper aerodigestive tract [16]. We modified this technique. We found that the mouse laryngeal anatomy was similar to that of humans and changed when the mouse throat was chemically manipulated.

LPRD develops slowly. In vivo, laryngeal mucosal changes are also slow. Shimazu et al. established a rabbit model of esophageal reflux to observe changes in laryngeal tissue [22] The laryngeal mucosal epithelium did not thicken significantly within 2 weeks of treatment but did after 8–12 weeks [22]. Asaoka et al. established a mouse model of laryngeal reflux; the laryngeal epithelial tissue of all mice was significantly thicker than that of controls 2 weeks after surgery [18]. Sasaki et al. established a mouse model of LPR; the laryngeal epithelium exhibited molecular and (early) histopathological alterations linked to neoplastic transformation after 45 days of feeding with gastroduodenal fluid [16]. In the present study, we found significant hyperplastic alterations in the mouse laryngeal epithelium after 45 days of feeding with artificial gastric juice containing pepsin.

The cited in vivo studies revealed that gastric contents damaged the laryngeal epithelium. Other works found that pepsin played an important role in this context [23]. Pepsin may induce the epithelial–mesenchymal transition (EMT) and inhibit mitochondrial function [10]. Johnson et al. found that pepsin induced the proliferation and growth of hypopharyngeal carcinoma FaDu cells and normal laryngeal epithelial cells 36 h after application and modulated the expression of carcinogenesis-related genes [11]. Also, pepsin induced vocal cord polyps by causing oxidative DNA damage [24]. Brief exposure to pepsin activated the expression of cancer-associated genes; a pathway analysis revealed a relationship between cancer and related signaling processes [25]. Therefore, chronic pepsin exposure could trigger laryngeal epithelial carcinogenesis [26]. Cells take up pepsin via receptor-mediated endocytosis; pepsin is stored in vesicles and transported to organelles such as the Golgi apparatus [2]. Pepsin absorbed into the laryngeal epithelium is inactive but stable because the mean pH of the laryngopharynx is 6.8. Inactivated pepsin is re-activated by even a slight decrease in pH; for example, a non-acidic reflux event. Reactivated pepsin damages laryngopharyngeal cells [2]. Pepsin (0.1 mg/mL) at pH 7 may also cause mitochondrial damage and triggers the expression of many genes and proinflammatory cytokines [11].

LPR causes laryngeal damage. Pepsin-containing refluxates trigger inflammation and other immune responses [13]. The Warburg effect is in play during the pathogenesis of inflammatory and precancerous lesions [26–28]. In premalignant Barrett esophagus (BE) cells, glycolysis and mitochondrial dysfunction gradually increased as normal BE cells progressed to esophageal adenocarcinoma [29]. During acute liver failure, the Warburg effect upregulates the levels of certain enzymes and metabolites (including lactate and GLUT-1), promoting inflammation [30]. We found that pepsin-containing artificial gastric juice promoted mouse laryngeal epithelial proliferation. Inhibition of Glut-1 expression by 2-DG suppressed the hyperplasia induced by elevated Glut-1 and H^+^/K^+^-ATPase α, β expression. Pepsin-containing artificial gastric juice promoted hyperplasia by upregulating Glut-1 and H^+^/K^+^-ATPase α, β expression; Glut-1 expression may modulate that of H^+^/K^+^-ATPase α, β. Pepsin induced mouse laryngeal epithelial hyperplasia by increasing Glut-1 expression, followed by H^+^/K^+^-ATPase α, β upregulation. We found a relationship between Glut-1 expression and H^+^/K^+^-ATPase α, β expression. We earlier showed that H^+^/K^+^-ATPase was present in normal laryngeal tissues and that expression was elevated in laryngeal carcinomas [7]. Stomach H^+^/K^+^-ATPase engages in acid secretion. Given the roles played by the Warburg effect in inflammation and precancerous lesions, we suggest that high-level H^+^/K^+^-ATPase α, β expression triggers H^+^ secretion that reduces the pH of the mouse laryngeal microenvironment, in turn re-activating pepsin that damages mitochondria, thus reprogramming glucose metabolism. McCormick et al. reported H^+^/K^+^-ATPase α, β expression in LPR laryngeal tissues, adjacent tissues, and laryngeal carcinomas. Hypopharyngeal carcinoma FaDu cells expressing H^+^/K^+^-ATPase α, β (ATP4A and ATP4B) induced mitochondrial damage and the expression of related genes [4]. H^+^ accumulation upregulated H^+/^K^+^-ATPase to maintain homeostasis by exporting H^+^. During high-level glycolysis, Glut-1 (encoded by a hypoxia-response gene) is overexpressed in inflamed tissues, enhancing glucose transport into cells, glycolysis, and lactate production [15]. The intracellular H^+^ of lactate accumulates, increasing the expression of the H^+^ transporters H^+^/K^+^-ATPase and H^±^ATPase, which shuttle H^+^ out of the cells to maintain homeostasis. The reduced microenvironmental pH further re-activates pepsin [31].

## Conclusions

Pepsin-containing artificial gastric juice promoted mouse laryngeal epithelial hyperplasia. Pepsin-induced changes in laryngeal epithelium were associated with abnormal expression of Glut-1 and H^+^/K^+^-ATPase α, β.

## Data Availability

Data are available on request to the authors.
